# Health Care Worker Contact with MERS Patient, Saudi Arabia

**DOI:** 10.3201/eid2012.141211

**Published:** 2014-12

**Authors:** Aron J. Hall, Jerome I. Tokars, Samar A. Badreddine, Ziad Bin Saad, Elaine Furukawa, Malak Al Masri, Lia M. Haynes, Susan I. Gerber, David T. Kuhar, Congrong Miao, Suvang U. Trivedi, Mark A. Pallansch, Rana Hajjeh, Ziad A. Memish

**Affiliations:** Centers for Disease Control and Prevention, Atlanta, Georgia, USA (A.J. Hall, J.I. Tokars, L.M. Haynes, S.I. Gerber, D.T. Kuhar, C. Miao, S.U. Trivedi, M.A. Pallansch, R. Hajjeh);; Dr. Soliman Fakeeh Hospital, Jeddah, Saudi Arabia (S.A. Badreddine);; Ministry of Health, Riyadh, Saudi Arabia (Z. Bin Saad, E. Furukawa, M. Al Masri, Z.A. Memish)

**Keywords:** MERS, coronavirus, healthcare, contact investigation, infection control, viruses, Middle East respiratory syndrome, Saudi Arabia

## Abstract

To investigate potential transmission of Middle East respiratory syndrome coronavirus (MERS-CoV) to health care workers in a hospital, we serologically tested hospital contacts of the index case-patient in Saudi Arabia, 4 months after his death. None of the 48 contacts showed evidence of MERS-CoV infection.

Middle East respiratory syndrome coronavirus (MERS-CoV) was initially isolated in September 2012 from a 60-year-old man from Bisha, Saudi Arabia (*1*). In June 2012, this index case-patient was hospitalized for severe respiratory disease in Jeddah at Dr. Soliman Fakeeh Hospital and subsequently died (*1*). As of November 3, 2014, a total of 897 laboratory-confirmed cases of MERS-CoV infection, including 325 deaths, had been reported to the World Health Organization; >85% of reported MERS-CoV cases and deaths have occurred in Saudi Arabia (*2*). The clinical syndrome among hospitalized MERS-CoV patients includes severe acute respiratory illness, sometimes associated with hypoxemic respiratory failure and extrapulmonary organ dysfunction (*3*); however, milder illness and asymptomatic infections have been identified through contact investigations (*4*–6). Transmission of MERS-CoV to health care workers (HCWs) has been reported (*5**,**6*), although no sustained community transmission has been identified.

A zoonotic origin of MERS-CoV has been hypothesized; camels potentially play a role in transmission (*7*), although the specific types of exposure associated with primary cases remain unknown. There remains a dearth of information on how MERS-CoV is spread and on transmission risks to HCW or other close contacts. Our objectives were to evaluate the degree and nature of HCW contact with the MERS-CoV index case-patient and to serologically assess HCWs for MERS-CoV infection. The field investigation was performed in October 2012; we awaited development and validation of MERS-CoV serologic assays before completing the study.

## The Study

The index case-patient was hospitalized on June 13, 2012, with a 7-day history of fever, cough, sputum expectoration, and shortness of breath. Precautions to prevent airborne transmission were taken by placing the patient in a private room with negative pressure for the first 2 days of hospitalization. After an infectious disease consultation on day 2, airborne-transmission precautions were replaced with droplet-transmission precautions; after a multidrug-resistant organism was isolated on day 4, contact-transmission precautions were implemented (*8*). The case-patient remained in a private room, under standard and contact-transmission precautions, throughout hospitalization until he died on day 11.

Using dates and units in which the case-patient received care, hospital staff initially identified HCWs who had had contact with the case-patient (came within 2 meters of the case-patient or his bedding, equipment, or body fluids). A comparison group of approximately equal numbers of HCWs (preferentially with similar job responsibilities) was selected from HCW who had had no known contact with the case-patient. Hospital infection control staff administered a brief, standardized questionnaire to both groups of HCWs. Information was collected on HCW demographics, job duties, and symptoms of respiratory disease during June 15–July 4, 2012, which corresponds to the period when the case-patient was hospitalized and an incubation period of 2–10 days, based on MERS-CoV natural history information available at the time of investigation. Specific information about circumstances of case-patient contact and potential for MERS-CoV exposure was collected from HCWs who had had contact with the case-patient. 

In October 2013 (4 months after the case-patient’s death), a blood specimen (<20 mL) was collected from each HCW and transported first to the Ministry of Health Western Regional Laboratory in Saudi Arabia and then to the US Centers for Disease Control and Prevention for MERS-CoV testing. Upon receipt, specimens were gamma-irradiated on dry ice and stored at −70°C. All specimens were tested by HKU5.2N nucleocapsid enzyme immunoassay (EIA) (*9*); incubations and substrate development were conducted at 37°C. MERS-CoV antibody positivity was defined as positive HKU5.2N screening EIA results and confirmed by MERS-CoV immunofluorescence or microneutralization assay (*9**,**10*); specimens with negative EIA results were considered antibody negative.

Of 56 HCWs identified as having had contact with the MERS-CoV case-patient, 5 were unavailable for interview and 3 refused serum collection, leaving 48 for the final analysis. Among HCW who had had case-patient contact, median age was 30.5 (range 22–57) years; 29 (60%) were female; 24 (50%) were nurses; 14 (29%) were physicians; 7 (15%) were respiratory technicians; and 1 each was a housekeeping, radiology, or infection control staff member. Six (13%) HCWs reported having a chronic medical condition (e.g., asthma, diabetes, hypertension), and 6 (13%) reported smoking tobacco. According to body mass index (BMI) calculations from self-reported height and weight, nearly half of HCWs were overweight (BMI 25.0–29.9, n = 11 [23%]) or obese (BMI ≥30.0, n = 12 [25%]).

Most of the 48 HCWs had reportedly come within 1 meter of the case-patient (89%); touched the case-patient (85%); or touched the case-patient’s bedding, equipment, or body fluids (62%) (Table). During a single shift, most (60%) HCWs reported <1 hour of case-patient contact, but 23% reported >5 hours of contact. HCWs reported having been present during airway suction (50%), nebulizer treatment (30%), sputum induction (23%), bronchoscopy (9%), and intubation (6%). Infection control precautions reportedly used by HCWs during contact with the case-patient included hand hygiene (100%) and/or wearing of gloves (94%), surgical mask (87%), and gown (40%); however, among those reporting use of these precautions, some admitted to <100% compliance and none used eye protection.

**Table:** Contact characteristics for 48 health care workers who had contact with Middle East respiratory syndrome coronavirus index case-patient, Jeddah, Saudi Arabia, June 2012

Characteristic	No. (%)
Unit where contact occurred	
Intensive care unit	21 (43.8)
Consult	7 (14.6)
Respiratory treatment	7 (14.6)
Respiratory disease unit	4 (8.3)
Emergency department	3 (6.3)
Bronchoscopy	2 (4.2)
Infectious disease unit	2 (4.2)
Virology laboratory	1 (2.1)
Infection control	1 (2.1)
Type of contact*	
Came within 1 m of patient	43 (89.6)
Touched patient	41 (85.4)
Touched patient's bedding, equipment, or body fluids	30 (62.5)
Collected clinical specimens	19 (39.6)
Emptied bedpan	11 (22.9)
Maximum contact duration during any shift	
<1 h	28 (58.3)
1–2 h	4 (8.3)
3–4 h	4 (8.3)
≥5 h	12 (25.0)
Care provided to patient*	
Physical examination	24 (50.0)
Assessment of vital signs	19 (39.6)
Lifting or positioning	15 (31.3)
Medication administration	14 (29.2)
Blood collection	14 (29.2)
Linen change	12 (25.0)
Bathing	11 (22.9)
Feeding	11 (22.9)
Intravenous catheter placement	11 (22.9)
Radiography	5 (10.4)
Present during medical procedures*	
Airway suction	24 (50.0)
Nebulizer treatment	14 (29.2)
Sputum induction	11 (22.9)
Bronchoscopy	4 (8.3)
Intubation	3 (6.3)
Infection control precautions used during patient contact*	
Hand hygiene	48 (100)
Gloves	45 (93.8)
Surgical mask	42 (87.5)
Gown	19 (39.6)
N95 mask	16 (33.3)
Eye protection	0

*Because >1 response could be selected for these characteristics, percentages may total >100%.

Respiratory disease symptoms during June 15–July 4 were reported by 10 (21%) HCWs who had had case-patient contact. Among these, symptoms included cough (40%), sore throat (30%), myalgia (30%), rhinorrhea (20%), self-reported fever (10%), diarrhea (10%), and sneezing (10%); 2 (20%) of these 10 HCWs sought medical care and received a diagnosis of pharyngitis. For comparison, respiratory symptoms were reported during the same period by 16 (33%) HCWs who had not had case-patient contact. Results of EIA testing of serum collected during October 14–17, 2012, from all 48 HCWs who had and all 48 who had not had case-patient contact were negative for MERS-CoV. Immunofluorescence assay of 13 randomly selected serum specimens also gave negative MERS-CoV results, supporting interpretation of EIA-negative specimens as antibody negative.

## Conclusions

This investigation provides indicators of transmission potential during the initial emergence of MERS-CoV. The lack of MERS-CoV transmission among HCWs in this study is similar to results of some published contact investigations (*11**,**12*) but different from others that reported transmission to HCW contacts (*5**,**6*). Recovery of cultivable virus from the sputum of the case-patient reported here (*1*) and the severity of illness suggest some potential for virus transmission. We did not assess use of recommended personal protective equipment during each episode of case-patient contact; however, the reported lack of use during every episode of patient contact suggests that some HCWs might have been exposed to MERS-CoV. Nonetheless, the infection control precautions that were used might have contributed to the demonstrated lack of transmission.

Serologic assays to determine past infections are useful for assessing the risk for MERS-CoV transmission (*13*). The assays used in this study have previously detected MERS-CoV–specific antibodies <2 weeks and >13 months after illness onset with >97% specificity (*9**,**14*). Although no severely ill HCWs were identified during this investigation, real-time reverse transcription PCR detection of MERS-CoV in respiratory tract specimens from asymptomatic or mildly ill HCW during other contact investigations has been described (*5**,**15*). The timing of this investigation 4 months after hospitalization of the case-patient precluded use of real-time reverse transcription PCR and potentially introduced recall bias during HCW interviews.

Despite numerous case-patient contact episodes among HCWs, we found no serologic evidence suggesting health care–associated transmission of MERS-CoV from the index case-patient. Rapid identification of potentially infected patients and implementation of infection control precautions can help protect HCWs. US Centers for Disease Control and Prevention recommendations for management of hospitalized patients with known or suspected MERS-CoV infection include implementing standard, contact, and airborne transmission precautions (*15*).
